# Biological properties of soluble CD146 and its role and mechanisms in disease progression

**DOI:** 10.3389/fimmu.2026.1892968

**Published:** 2026-07-20

**Authors:** Juan Meng, Qi Deng, Shu-qiong Xu, Ping Chen, Dan-Ping Huang, Shengfeng Zeng, Juan Huang

**Affiliations:** 1 Department of Pediatrics, West China Hospital Sichuan University Jintang Hospital. Jintang First People’s Hospital, Chengdu, China; 2 Department of Gastroenterology, West China Hospital Sichuan University Jintang Hospital. Jintang First People’s Hospital, Chengdu, China

**Keywords:** biomarker, clinical translation, soluble CD146, therapeutic target, tumor

## Abstract

Soluble CD146 (sCD146) is a key circulating biomarker released from vascular endothelial cells via enzymatic cleavage under conditions of stress or activation.sCD146 not only dynamically reflects the integrity of the microvascular endothelial barrier but also profoundly drives the malignant progression of multisystem diseases through targeted interactions with its receptors (such as angiopoietin AMOT and VEGFR2). In malignant solid tumors, it accelerates tumor invasion and immune evasion by inducing epithelial-mesenchymal transition (EMT) and cancer stem cell (CSC) phenotypes; In cardiovascular diseases and early-life developmental abnormalities (such as bronchopulmonary dysplasia), it serves as a highly sensitive marker of tissue stasis and microvascular stress. However, the clinical application of sCD146 currently faces substantial translational hurdles: on the one hand, the lack of a standardized, cross-platform detection system and universally accepted clinical-pathological cutoff values hinders data interoperability across centers; on the other hand, its baseline expression exhibits heterogeneity in complex complications, and whether it serves as a “key mediator” of disease progression or a “bystander” of concomitant injury remains inconclusive in certain pathological states. In light of this, this review breaks down disciplinary barriers to systematically summarize the latest medical advances regarding sCD146 across oncology, cardiovascular, neuroimmunology, and reproductive development fields. It clarifies the core pathogenic mechanisms of sCD146 in different microenvironments, addresses practical challenges in clinical translation, and provides a solid theoretical foundation to advance sCD146 from a laboratory biomarker to a clinical precision diagnostic and therapeutic target.

## 1 Introduction

CD146 (also known as MUC18 or MCAM), a transmembrane glycoprotein of the immunoglobulin superfamily (1), has traditionally been widely considered merely a structural adhesion molecule localized exclusively at vascular endothelial junctions. This intrinsic morphological state, strictly anchored physically to the plasma membrane or intercellular junctions, is defined as membrane-bound CD146 (mCD146).However, groundbreaking research in recent years has revealed that under pathological stressors such as hypoxia, mechanical stretching, or inflammatory storms, the extracellular domain of CD146 can be released into the bloodstream through enzymatic cleavage mediated by matrix metalloproteinases (e.g., ADAM10/TACE) or via alternative splicing of mRNA, forming sCD146 (2). More importantly, numerous *in vitro* and *in vivo* experiments have confirmed that sCD146 is not merely a “metabolic byproduct” passively released following endothelial damage, but rather a highly bioactive, multifunctional signaling molecule. It can interact with its homologous receptors (such as VEGFR2, angiomotin, and the integrin family) via paracrine or endocrine mechanisms, actively driving pathological angiogenesis, epithelial-mesenchymal transition (EMT), and the transendothelial migration of pro-inflammatory cells (3, 4).

As the underlying mechanisms continue to be elucidated, the clinical translational value of sCD146 is becoming increasingly evident. Whether in predicting early resistance to anti-angiogenic drugs, distinguishing between heart failure-induced dyspnea and tissue-specific congestion, or monitoring developmental defects in the early-life brain-placental axis, sCD146 demonstrates unique potential superior to traditional biomarkers. However, its transition from the laboratory to routine clinical testing still faces significant challenges: on the one hand, differences in antibody clone numbers across different testing platforms make it difficult to establish a unified clinical reference cutoff value across centers (5); on the other hand, in complex complications involving multisystem involvement, whether sCD146 is the “key mediator” of disease progression or merely a “bystander” accompanying microvascular remodeling (6) remains a contentious issue urgently requiring clarification in the field of translational medicine.

Addressing this unresolved clinical challenge, this review breaks away from the traditional single-discipline framework and, for the first time, systematically constructs a multidimensional pathogenic network of sCD146 spanning malignant tumors, cardiovascular circulation, neuroimmune inflammation, and reproductive and developmental medicine. This article will conduct an in-depth analysis of the core pathogenic mechanisms of sCD146 in different pathological microenvironments, objectively evaluate its translational potential and limitations across large-scale clinical cohorts, and prospectively explore precision intervention strategies targeting this signaling axis (such as the neutralizing monoclonal antibodies M2J-1 and Mucizumab).We aim to provide theoretical support and future research directions from a holistic perspective to bridge the translational gap between sCD146’s “basic discovery” and its establishment as a “clinical diagnostic and therapeutic target”.

## 2 Biological characteristics of sCD146

### 2.1 Definition and origin

sCD146 is the primary free form of the transmembrane receptor CD146 in the circulatory system and is increasingly emerging as a cutting-edge biomarker in the field of precision vascular medicine. It is worth emphasizing that sCD146 has challenged the conventional perception of it as a “cell detachment metabolite” and has been redefined as an effector molecule with independent signaling capabilities and high biological activity.

### 2.2 Mechanisms of production and release

The generation of sCD146 primarily occurs through two precise biochemical pathways: enzymatic shedding and alternative splicing (2).

Shedding: CD146 on the cell membrane surface is cleaved by specific matrix metalloproteinases (such as ADAM-10 and TACE), thereby releasing it into the extracellular space. Studies have shown that the influx of calcium ions Ca^2+^into endothelial cells is a key signal triggering the shedding and release of CD146.Furthermore, inflammatory cytokines (such as TNF-α) and pathophysiological stimuli such as vascular endothelial stretch and oxidative stress can also significantly upregulate the release of sCD146.

Alternative Splicing: Alternative splicing of the primary transcript directly generates different forms of sCD146 variants, namely I10-sCD146, which retains intron 10, and I5-13-sCD146, which retains introns 5–13.

**Figure 1:**
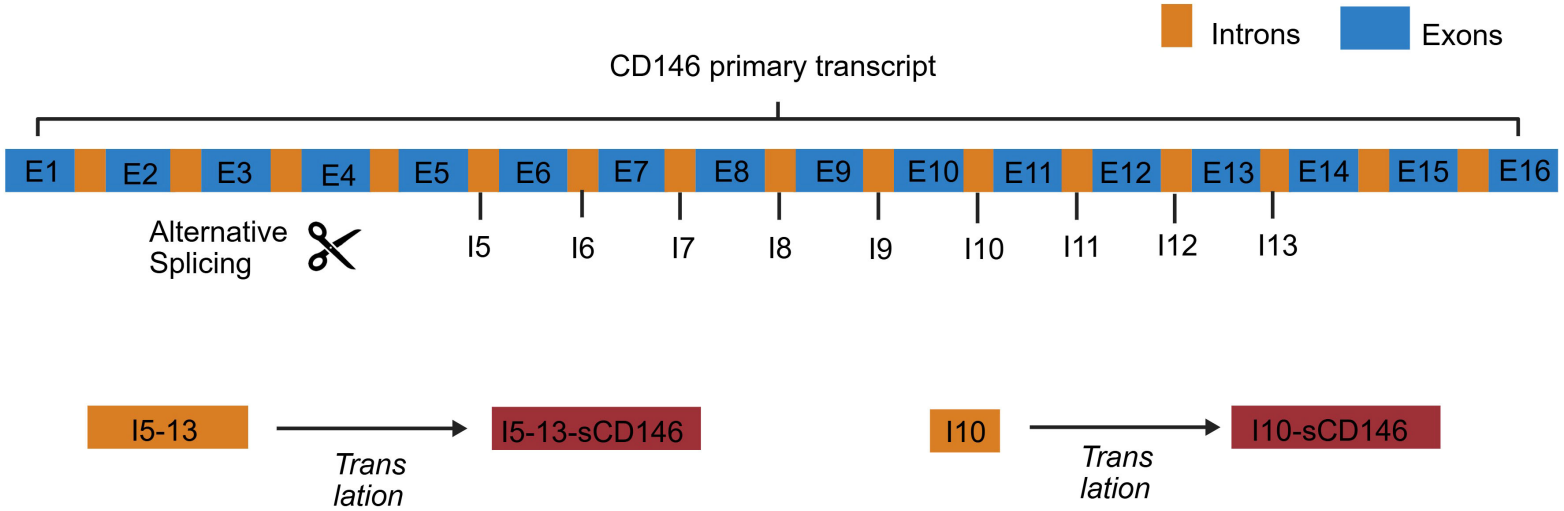
Alternative splicing and translation mechanism of sCD146 variants. The CD146 primary transcript consists of alternating exons (blue blocks) and introns (orange blocks). Through alternative splicing, transcripts retaining specific intron sequences (e.g., I5–13 and I10) are generated. Following translation, these transcripts produce free-floating protein variants (I5-13-sCD146 and I10-sCD146) lacking a transmembrane domain, which exert specific biological functions in pathological microenvironments.

To illustrate these two distinct molecular processes more clearly, **Supplementary Figure 1** and **Figure 1** depict the detailed mechanisms of proteolytic shedding and alternative splicing of the primary transcript, respectively.

### 2.3 Physiological functions

#### 2.3.1 Maintenance of vascular endothelial homeostasis and barrier integrity

mCD146 is continuously expressed at endothelial junctions and participates in maintaining endothelial monolayer adhesion and vascular permeability (7, 8). As a circulating derivative, sCD146 reflects the structural integrity of endothelial junctions and regulates endothelial barrier function. Under physiological conditions, sCD146 maintains low concentrations in plasma, contributing to the stabilization of vascular permeability and preventing excessive fluid leakage from blood vessels; simultaneously, it modulates the distribution of endothelial cytoskeletal proteins and focal adhesion molecules, supporting endothelial homeostasis and vascular turgor (9).

#### 2.3.2 Promotion of physiological angiogenesis and endothelial progenitor cell function

As an angiogenic factor, sCD146 plays a crucial role in physiological angiogenesis (10, 11). It chemotaxes endothelial cells and endothelial colony-forming cells (ECFCs), promotes endothelial cell migration, proliferation, and lumen formation, and enhances the angiogenic capacity of endothelial progenitor cells. Mechanistically, sCD146 upregulates VEGFR2 expression, activates eNOS, and synergizes with VEGF to exert an additive pro-angiogenic effect, supporting vascular growth and repair during tissue development and ischemia (12). In animal models of hindlimb ischemia, sCD146 promotes neovascularization, improves tissue perfusion, and reduces necrosis and fibrosis, demonstrating its physiological significance in vascular regeneration (13–15).

#### 2.3.3 Regulation of placental development and the biological behavior of trophoblast cells

During normal pregnancy, sCD146 is dynamically expressed and participates in the regulation of placental vascular development and trophoblast function. Under physiological conditions, sCD146 levels gradually decline as pregnancy progresses, facilitating the migration and invasion of extra-villous trophoblast cells, spiral artery remodeling, and normal placental implantation. By interacting with galectin-1 (Gal-1) and the VEGFR2 signaling pathway, sCD146 regulates trophoblast migration and invasion. As a physiological regulator, it maintains appropriate trophoblast activity and the process of placental vascular morphogenesis, ensuring normal embryonic implantation and placental development (16).

#### 2.3.4 Regulation of leukocyte transendothelial migration and immune cell homeostasis

sCD146 participates in physiological immune surveillance by regulating leukocyte transendothelial migration. It specifically binds to monocytes, promoting their moderate transendothelial migration, which is crucial for tissue immune surveillance and inflammatory homeostasis (17). Furthermore, sCD146 serves as a phenotypic marker for human Th17 lymphocytes. Under physiological conditions, sCD146 participates in regulating Th17 cell localization and function, maintaining the balance of immune subsets without triggering excessive inflammatory activation (18, 19).

#### 2.3.5 Supporting the stemness and regenerative capacity of mesenchymal stem cells

sCD146 is closely associated with the biological characteristics of mesenchymal stem cells (MSCs). sCD146-positive MSCs constitute a functional subset characterized by enhanced stemness, proliferative capacity, and immunosuppressive potential.sCD146 maintains the stem cell phenotype of MSCs, inhibits cellular senescence, enhances MSC survival and paracrine functions, and promotes tissue repair. *In vitro* experiments show that sCD146 increases the colony-forming efficiency of ECFCs, prolongs the regenerative activity of endothelial progenitor cells, and plays a significant role in physiological vascular maintenance and tissue regeneration (20).

#### 2.3.6 Involvement in tissue development and organ homeostasis

Under physiological conditions, CD146 and sCD146 are extensively involved in multi-organ development. In the thymus, CD146 is expressed on cells in the thymic microenvironment and participates in T-lymphocyte development and the structural integrity of the thymus; in blood vessels and connective tissue, sCD146 coordinates angiogenesis and extracellular matrix remodeling, maintaining normal tissue structure and function. Overall, sCD146 acts as a physiological regulator linking endothelial function, cell migration, angiogenesis, and immune homeostasis (21, 22).

### 2.4 sCD146: a molecular hub for microenvironmental communication

Compared to established markers of endothelial dysfunction such as VCAM-1 and ICAM-1—which primarily serve as indicators of generalized endothelial activation and leukocyte adhesion driven by inflammatory cascades—sCD146 plays a fundamentally different role. While soluble VCAM-1 and ICAM-1 are largely passive byproducts of vascular inflammation, sCD146 directly reflects the physical breakdown of endothelial junctional integrity under severe mechanical or hypoxic stress. More importantly, sCD146 acts as an active, multi-directional signaling driver that orchestrates irreversible vascular remodeling, pathological angiogenesis, and EndoMT, thereby transitioning from a mere ‘bystander’ of endothelial injury to a key mediator of disease progression.

The pathological significance of sCD146 has far surpassed its initial recognition as a “marker of endothelial injury. “Triggered by various pathological stresses, free sCD146 acts as a highly active, multifunctional signaling molecule that penetrates the extracellular matrix to enter the microenvironments of different target organs. It not only interferes with normal protein interactions through “competitive ligand inhibitor” but also directly activates target cell membrane receptors, inducing profound changes including cytoskeletal reorganization, metabolic reprogramming, and transcriptional phenotypic transformation. These core molecular events drive distinctly different pathological outcomes across various target organs (see Section 2 for details). These complex processes of extracellular matrix penetration, ligand competition, and transcriptional reprogramming constitute the underlying logic by which sCD146 regulates disease progression; the specific core molecular mechanisms and targeted interaction pathways are summarized in **Table 1**.

**Table 1:** Pathophysiological functions and molecular mechanisms of soluble CD146.

Core pathological mechanisms	Associated diseases	Target cells/interaction partners	Key receptors or binding partners	Core downstream signaling pathways	Ultimate biological effects	References
Epithelial-Mesenchymal Transition (EMT & Metastasis)	Melanoma, Non-Small Cell Lung Cancer (NSCLC)	Solid tumor cells (paracrine/autocrine)	Tumor cell surface receptors	Wnt/β-catenin signaling pathway, NF-κB	Upregulates vimentin, downregulates E-cadherin; cells lose polarized junctions and acquire the ability to migrate distally through the basement membrane	(23–25)
Pathological angiogenesis (Angiogenesis)	Glioblastoma (GBM), breast cancer, peripheral artery disease (PAD)	Endothelial cells (EC)	AMOT (Angiopoietin), in conjunction with VEGFR2	PI3K/AKT signaling pathway, p38 MAPK	promotes endothelial cell polarization, proliferation, and migration, and induces microvascular lumen formation (establishment of compensatory blood supply)	(10, 26–29)
Leukocyte transendothelial migration (TEM & barrier disruption)	Multiple sclerosis (MS), anti-NMDAR encephalitis	Peripheral autoreactive T cells, monocytes	Specific ligands/integrins on the surface of immune cells	Src/FAK kinase activation - RhoA/ROCK cytoskeletal reorganization	Induces VE-cadherin phosphorylation and endocytosis, facilitating the widening of intercellular gaps and the transmigration of immune cells across the blood-brain barrier (BBB)	(30–33)
“Competitive Ligand Inhibitor” effect	Preeclampsia (PE), recurrent miscarriage (RM)	Migratory signaling molecules in their free state	Gal1 (galectin-1)	Blocks the normal interaction between Gal1 and receptors on extra-villous trophoblast (EVT) cells	Competitively blocks Gal1, leading to loss of migratory capacity in EVTs and failure of uterine spiral artery remodeling	(34–36)
Endothelial-smooth muscle crosstalk (Vascular Remodeling)	Pulmonary Arterial Hypertension (PAH)	Pulmonary Artery Smooth Muscle Cells (PASMCs)	Integrin αvβ3, trans-activates EGFR	MAPK/ERK survival pathway, synergistically with HIF-1α, drives the Warburg effect	Loss of contractile markers (α-SMA↓) and acquisition of a proliferative/anti-apoptotic phenotype (Bcl-2/Bax imbalance, collagen/MMPs↑), leading to irreversible medial thickening	(37)
Endothelial-Mesenchymal Transition (EndoMT & Fibrosis)	Systemic sclerosis (SSc)	Endothelial cells themselves (autocrine cycle)	Responds to the specific variant I5-13-sCD146 via its own membrane receptor	Wnt-1/β-catenin promotes fibroblast differentiation, synergizing with the TGF-β/Smad pathway	Loss of endothelial markers (VE-cadherin↓), acquisition of mesenchymal characteristics (α-SMA↑), complete transformation into myofibroblasts, driving massive collagen deposition in tissues	(38–40)

## 3 Association of sCD146 with disease

**Figure 2:**
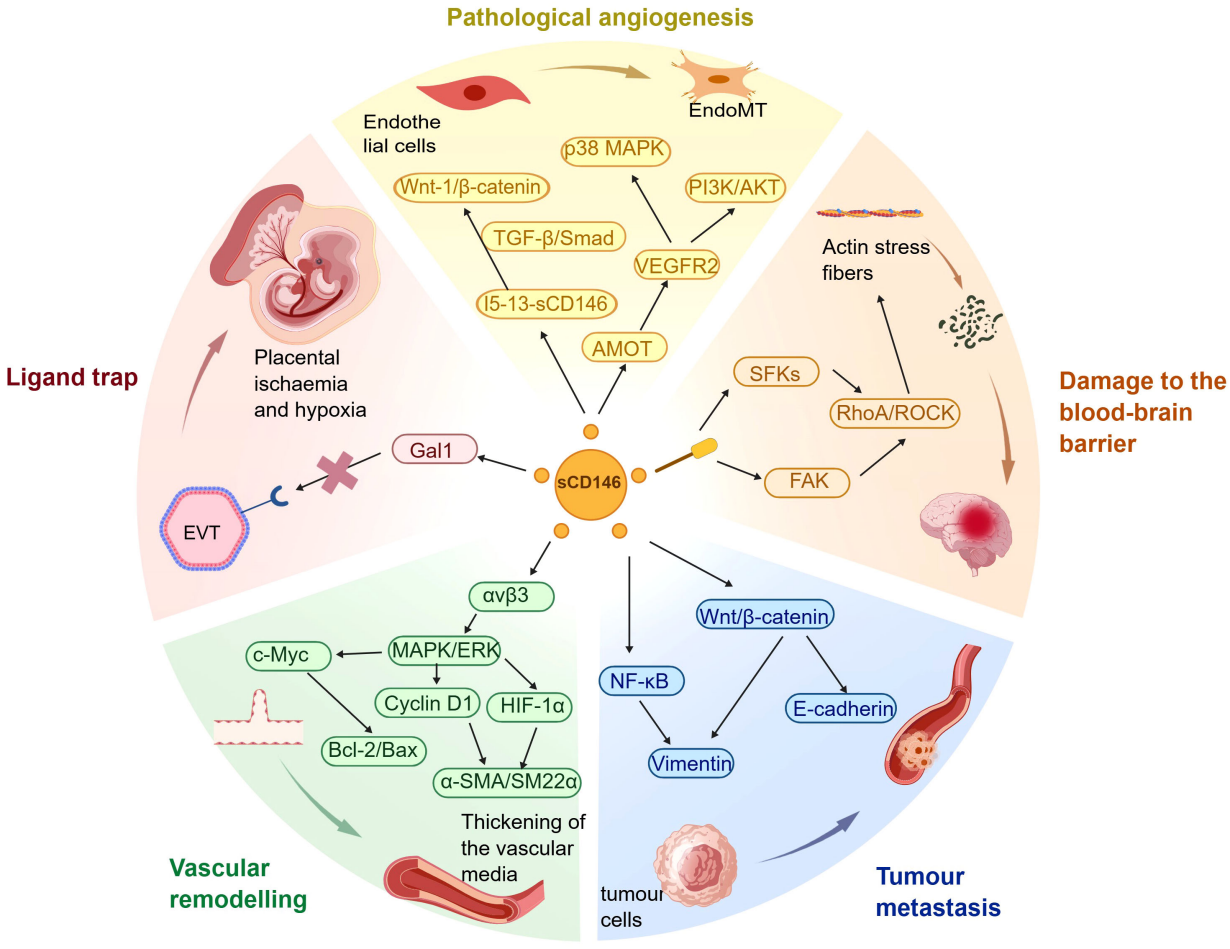
Schematic panorama of the core molecular mechanisms in sCD146-mediated multi-systemic diseases. As a pleiotropic signaling hub, free sCD146 drives specific pathological progression via receptor crosstalk across diverse target organ microenvironments: (1) Pathological angiogenesis and EndoMT (Top, yellow): sCD146 binds to AMOT/VEGFR2 to activate PI3K/AKT and p38 MAPK pathways, promoting angiogenesis; its variant I5-13-sCD146 induces endothelial-to-mesenchymal transition (EndoMT) via the Wnt-1/β-catenin and TGF-β/Smad axes. (2) Damage to the blood-brain barrier (Right, orange): It triggers actin stress fiber rearrangement by activating FAK and SFKs-RhoA/ROCK pathways, leading to BBB disruption. (3) Tumor metastasis (Bottom right, blue): In tumor cells, it activates Wnt/β-catenin and NF-κB pathways to upregulate vimentin and downregulate E-cadherin, inducing epithelial-mesenchymal transition. (4) Vascular remodeling (Bottom left, green): Binding to integrin αvβ3 activates MAPK/ERK and HIF-1α signaling, driving the thickening of the vascular media. (5) Competitive ligand inhibitor (Left, red): Under placental ischemia and hypoxia, sCD146 acts as a decoy to competitively bind Gal1, blocking its normal interaction with extravillous trophoblasts (EVT) and leading to pathological pregnancies.

When the body experiences ischemia, hypoxia, mechanical traction, or immune attack, the endothelial barrier is compromised, leading to the massive release of sCD146 into the bloodstream. As a highly active, multifunctional signaling molecule, sCD146 interacts with its receptor network to exert specific pathogenic effects in different pathological microenvironments. Based on this molecular foundation, the pathogenic effects of sCD146 in clinical settings exhibit high systemic heterogeneity and target-organ specificity. Before delving into its specific manifestations in different diseases, we first constructed a schematic overview of the core molecular mechanisms underlying sCD146-mediated multisystemic diseases (as shown in **Figure 2**), aiming to visually illustrate how it drives tissue damage and malignant remodeling—from the brain, heart, and lungs to the maternal-fetal interface—under various pathological stresses such as hypoxia-ischemia, mechanical stretching, or immune storms.

### 3.1 Malignant solid tumors

Highly active angiogenesis and tissue infiltration in the tumor microenvironment make sCD146 a malignant catalyst for various solid tumors.

#### 3.1.1 Melanoma

Melanoma cells release large amounts of sCD146 into the extracellular space via the highly expressed ADAM10/TACE protease (41). This free protein not only acts as a chemokine to attract vascular endothelial cells, but more importantly, it binds to receptors on the surface of tumor cells via an autocrine pathway, activating the Wnt/β-catenin signaling pathway. This process potently induces the epithelial-mesenchymal transition (EMT) in tumor cells, causing them to lose tight junctions and acquire the ability to penetrate the basement membrane (23, 24). This explains why melanoma patients with extremely high circulating sCD146 concentrations often develop distant hematogenous and lymphatic metastases at an earlier stage (42–46).

#### 3.1.2 Glioblastoma

GBM is one of the most highly vascularized tumors in the human body (26). By acting synergistically with VEGFR2, sCD146 is strongly associated with abnormal microvascular development (MVD) within the tumor. In clinical translation, the most critical mechanism lies in “therapeutic escape”: when the VEGF pathway is blocked using bevacizumab (an anti-VEGF monoclonal antibody), tumor cells compensatorily secrete more sCD146 to maintain blood supply by activating alternative angiogenesis pathways (such as the AMOT signaling axis).Consequently, a “rebound” increase in serum sCD146 has become a clinical warning sign for monitoring resistance to anti-angiogenic drugs (27).

#### 3.1.3 Breast cancer

In triple-negative breast cancer (TNBC), sCD146 secreted by tumor and stromal cells enters the microenvironment and induces tumor cells to upregulate cancer stem cell (CSC) markers such as CD44 and Sox2, enhancing their self-renewal and resistance to chemotherapy (28). Preclinical evidence suggests that this mechanism may drive an expanded fraction of cancer stem cells among tumor cell populations, which augments tumor self-renewal potential and confers resistance to conventional chemotherapies such as taxanes. Furthermore, sCD146-mediated endothelial activation creates a permissive vascular pathway facilitating breast cancer metastasis to bone and lung (29, 47).

#### 3.1.4 Non-small cell lung cancer and hepatocellular carcinoma

Both of these tumor types are highly invasive and have a rich blood supply. Here, sCD146 primarily reflects the tumor’s overall angiogenic burden. As TNM staging progresses, tumor tissue not only promotes local angiogenesis via sCD146 but also indicates widespread fragility of the endothelial barrier by elevating circulating levels. The statistically independent prognostic significance (reduced OS/PFS) is precisely based on the dual pathological logic that sCD146 simultaneously reflects both the tumor’s “volumetric burden” and its “potential for distant invasion. “In the progression of HCC, sCD146 accelerates malignant transformation against a background of cirrhosis by driving the transformation of hepatic stellate cells into myofibroblasts, thereby enhancing the sensitivity of early diagnosis in conjunction with AFP (25, 48).

#### 3.1.5 Clear cell renal cell carcinoma

ccRCC is characterized by VHL gene mutations leading to excessive activation of the HIF (hypoxia-inducible factor) pathway. sCD146 is one of the key downstream factors regulated by HIF. When treated with TKIs such as sunitinib, if the hypoxic state within the tumor fails to improve due to vascular inhibition and instead stimulates the release of higher levels of sCD146, this indicates that the tumor is establishing new collateral circulation by upregulating sCD146 to counteract hypoxia. Therefore, sCD146 is an important molecular marker for assessing TKI treatment sensitivity and tissue oxygenation status. Clinically, patients with plasma sCD146 variability below 120% after the first treatment cycle typically exhibit longer progression-free survival (PFS) (49).

#### 3.1.6 Osteosarcoma

As the most common primary highly malignant bone tumor, osteosarcoma is characterized by an abnormally rich microvascular network and a tendency for very early hematogenous lung metastasis. In the osteosarcoma microenvironment, tumor cells not only highly express transmembrane CD146 but also shed and release large amounts of sCD146.Circulating sCD146 exerts a potent angiogenic driving force, establishing dense vascular channels that enable tumor cells to breach the cortical bone and invade the peripheral blood circulation. Concurrently, sCD146 directly acts on osteosarcoma cells via autocrine/paracrine mechanisms, significantly enhancing their potential for distant migration and invasion. Multiple clinical transcriptomic and large-scale cohort studies have confirmed that high levels of sCD146 expression are closely associated with a sharp increase in the risk of early metastasis and a significant reduction in overall survival (OS) in osteosarcoma patients. Therefore, sCD146 is not only an independent adverse prognostic marker for assessing the risk of osteosarcoma metastasis but also a highly promising molecular target for future exploration of novel radioimmunotherapy or targeted monoclonal antibody blockade (50).

A comprehensive summary of the clinical translation significance and targeted interventions for sCD146 in malignant solid tumors is provided in **Table 2A**.

**Table 2A:** Summary of sCD146 associations, clinical trials, and interventions in malignant solid tumors.

Disease category	Disease name	Core molecular mechanism	Clinical translation sgnificance	Clinical trials (cohort size/strength of evidence)	Potential interventions/target drugs	References
Malignant Solid Tumors	Melanoma	Induces Angiomotin overexpression, activates the Wnt/β-catenin axis to promote EMT.	Key indicator of tumor burden and risk of distant metastasis.	Prospective cohort: Dynamic monitoring of stage IV patients; sCD146 is positively correlated with the number of metastatic lesions.	M2J-1 monoclonal antibody; humanized monoclonal antibody Mucizumab.	(23, 24, 41–46)
	Glioblastoma (GBM)	Integrin αvβ3 mediates invasion; anti-VEGF therapy induces compensatory hypersecretion.	The “first signal” predicting resistance to anti-angiogenic drugs.	Experimental cohort: Analysis of serum from GBM patients; validation of resistance mechanisms using xenograft models.	Treatment with mucizumab in combination with bevacizumab.	(26, 27)
	Triple-negative breast cancer (TNBC)	Paracrine upregulation of CSC (cancer stem cell) markers.	Identification of subgroups with extremely high metastatic potential; assessment of malignancy.	Prospective trial: involving 160 patients, with a significantly increased risk of recurrence in the high-expression group.	M2J-1 mAb.	(28, 29, 47)
	Clear cell renal cell carcinoma (ccRCC)	Regulated by the HIF pathway; antagonizes sunitinib-induced cytotoxicity.	Assessment of sensitivity to targeted therapies and tissue oxygenation status.	Multicenter prospective study: First-cycle variability <120% predicts longer PFS.	Targeted therapy in combination with sunitinib.	(49)
	Non-small cell lung cancer (NSCLC)	Reflects tumor microvascular burden and endothelial integrity.	A prognostic factor independent of staging that predicts overall survival (OS).	Clinical observation: Follow-up of 52 advanced-stage patients showed that high baseline levels were associated with poor survival.	Endothelial protection combined with chemotherapy.	(48)
	Hepatocellular Carcinoma (HCC)	Drives hepatic stellate cell activation and mediates a pro-fibrotic microenvironment.	Aids in distinguishing cirrhosis from early-stage cancer using AFP.	Clinical comparative study: 120 HCC patients vs. cirrhosis vs. control group.	Anti-signal intervention targeting the liver microenvironment.	(25)
	Osteosarcoma	Drives lethal microvascular angiogenesis networks and directly enhances tumor cell migration and invasion potential through autocrine/paracrine mechanisms to breach the cortical bone	High expression is closely associated with a sharp increase in the risk of early hematogenous lung metastasis and a significant shortening of overall survival (OS), making it an independent marker of poor prognosis.	Confirmed by clinical transcriptomics and large-to-medium-sized observational cohorts/Evidence strength: Grade B/C	Targeted neutralizing antibodies; explored as a direct target for radioimmunotherapy	(50)

### 3.2 Cardiovascular and circulatory system diseases

#### 3.2.1 Heart failure

Venous stasis caused by heart failure leads to a sharp increase in pressure in the peripheral and visceral venous beds (51, 52). This sustained hemodynamic overload and mechanical stretch trigger transient calcium (Ca^2+^) influx into vascular endothelial cells, which in turn strongly activates matrix metalloproteinases (MMPs) on the cell membrane surface, such as ADAM10 and TACE (53). These activated enzymes precisely cleave the extracellular domain of transmembrane CD146, leading to massive shedding of sCD146 into the bloodstream (54). Therefore, sCD146 in heart failure is not a product of myocardial injury, but rather a direct biochemical reflection of “the venous system undergoing immense physical tension. “Large clinical cohorts such as LEDAs and MEDIA-DHF have confirmed that sCD146 is a highly specific surrogate marker of tissue congestion, capable of precisely guiding diuretic titration and volume-reduction therapy (55–62).

#### 3.2.2 Acute ischemic stroke

During the acute phase of cerebral infarction, the local cerebral vasculature suffers severe ischemic and hypoxic damage, leading to acute oxidative stress and even necrosis of cerebral microvascular endothelial cells. At this stage, the release of sCD146 not only serves as a marker of physical endothelial rupture, but free, high-concentration sCD146 also acts as a pro-inflammatory chemokine, mediating the migration of monocytes from the peripheral circulation across the damaged blood-brain barrier (BBB) into the brain parenchyma, triggering a secondary inflammatory cascade in the ischemic penumbra, and thereby expanding the infarct volume (63).

#### 3.2.3 Lower limb/peripheral artery disease

In the microenvironment of chronic lower limb tissue ischemia caused by PAD, sCD146 exhibits classic “double-edged sword” characteristics. Ischemia first acts as a stressor to induce the release of sCD146;but subsequently, this released sCD146 binds to and activates the VEGFR2/AMOT signaling axis on endothelial colony-forming cells (ECFCs) via paracrine action. This activation strongly stimulates endothelial cell proliferation and tubulogenesis, attempting to establish collateral circulation in the ischemic region, serving as an important endogenous compensatory mechanism for the body to counteract chronic ischemia (10).

#### 3.2.4 Atherosclerosis

The formation of atherosclerotic plaques begins with impaired endothelial function. Lipid deposition and abnormal shear stress lead to the sustained release of sCD146 by endothelial cells (64). More critically, sCD146 increases the local adhesion of the vascular wall, specifically recruiting pro-inflammatory monocytes (macrophage precursors) from peripheral blood to adhere to and infiltrate the subendothelial space (65). After phagocytosing lipids, these cells form foam cells, accelerating the expansion of the lipid core within the plaque and the thinning of the fibrous cap. This associative finding suggests a potential mechanism by which sCD146 contributes to plaque instability, though further *in vivo* causal validation remains to be established (18, 37, 66–68).

#### 3.2.5 Pulmonary hypertension

The critical hallmark of pulmonary hypertension is the progressive narrowing and remodeling of pulmonary arterioles. Under persistent stimulation by chronic hypoxia, inflammation, or abnormal blood flow shear stress, pulmonary microvascular endothelial cells exhibit an abnormal state of “hyperactivation” and proliferation, accompanied by massive shedding of sCD146.In the pulmonary vascular microenvironment, these free sCD146 molecules exert potent paracrine effects: they diffuse downward and directly act on underlying pulmonary arterial smooth muscle cells (PASMCs), activating downstream mitogenic signaling pathways. Preclinical models indicate that this cross-cellular signaling axis contributes to the transition of smooth muscle cells from a quiescent state to a proliferative “synthetic” phenotype. This ultimately leads to extreme thickening of the pulmonary microvascular media, centripetal narrowing of the lumen, and even the formation of characteristic “plexiform lesions.” Therefore, elevated sCD146 in this context is no longer merely a sign of congestion, but directly reflects and drives the malignant anatomical remodeling associated with the progressive increase in pulmonary vascular resistance (30).

The clinical trials and interventional strategies targeting sCD146 in cardiovascular diseases are summarized in **Table 2B**.

**Table 2B:** Summary of sCD146 associations, clinical trials, and interventions in the cardiovascular and circulatory system.

Disease category	Disease name	Core molecular mechanism	Clinical translation significance	Clinical trials (cohort size/strength of evidence)	Potential interventions/target drugs	References
Cardiovascular and Circulatory System	Acute/Chronic Heart Failure	Responsive to mechanical stretching of venous pressure; ADAM10/TACE-mediated shedding.	A specific indicator of tissue congestion, superior to volume markers.	LEDA cohort (n=437); MEDIA-DHF cohort (n=146).	Sacubitril/valsartan.	(51–62)
	Carotid atherosclerosis	Activates the MMP-9/IL-6 axis, driving plaque instability.	Assessment of vulnerable plaque risk and the stroke prevention window.	Prospective study: Involving 40 surgical patients, sCD146 was positively correlated with intraluminal neovascularization.	Statins/Inflammation inhibition.	(18, 37, 64–68)
	Peripheral Arterial Disease (PAD)	Combined VEGFR2/AMOT activation of eNOS promotes EPC survival.	Assessment of the body’s endogenous collateral circulation repair capacity.	Functional validation: EPC pretreatment and reinfusion experiment in PAD patients.	Local injection of recombinant sCD146 protein.	(10)
	Acute ischemic stroke (AIS)	Reflects BBB disruption and vasculitis following ischemia-reperfusion.	Assessment of stroke lesion depth and risks associated with reperfusion therapy.	Observational study: Strong correlation between admission NIHSS score and sCD146 levels.	Endothelial protectants (e.g., edaravone).	(63)
	Pulmonary hypertension (PH)	Acts on smooth muscle EGFR, inducing synthetic transformation and hypertrophy.	Indicates progressive increases in pulmonary vascular resistance and anatomical remodeling.	Comparative analysis: 25 patients with PAH and CTEPH versus a serological control group.	MAPK/ERK inhibitor.	(30)

### 3.3 The nervous system and autoimmune inflammation

The ability of sCD146 to mediate leukocyte transendothelial migration enables it to play a key role in barrier disruption triggered by immune inflammation.

#### 3.3.1 Multiple sclerosis

During acute relapses of MS, microvascular endothelial cells in the central nervous system (CNS) undergo enzymatic shedding of the ADAMs family under the stimulation of inflammatory factors, leading to a sharp increase in sCD146 concentrations in cerebrospinal fluid (31).Free sCD146 exhibits potent chemotactic activity: it can specifically bind to autoreactive T cells (particularly Th17 cells) and pro-inflammatory monocytes in the peripheral circulation that express the corresponding receptor. Current evidence suggests that sCD146 facilitates the targeted adhesion and subsequent transmigration of these immune cells across the normally tight blood-brain barrier (BBB) into the brain parenchyma. This loss of “gating” control directly leads to immune attacks on myelin, the severity of which is significantly positively correlated with the Expanded Disability Status Scale (EDSS) and serves as a sensitive biochemical indicator for assessing BBB integrity (32, 33).

#### 3.3.2 Anti-NMDAR encephalitis

Although the core pathogenesis of anti-NMDAR encephalitis involves antibody-mediated synaptic damage, the activation of local vascular endothelium is a prerequisite for antibody entry into the CNS. Elevated levels of sCD146 in cerebrospinal fluid reflect the initial response of intracerebral microvessels to the inflammatory storm. With the initiation of immunosuppressive therapy (such as plasmapheresis or corticosteroid treatment), the systemic and intracerebral inflammatory burden is reduced, the vascular endothelium stabilizes, and the release of sCD146 consequently decreases. Therefore, fluctuations in sCD146 concentrations can provide a real-time reflection of the activity of intracerebral inflammation, serving as an important auxiliary tool for clinically assessing treatment efficacy and determining whether to reduce medication dosage (38).

#### 3.3.3 Systemic sclerosis

SSc is characterized by recurrent microvascular damage and occlusion. Significantly elevated levels of sCD146 (particularly the I5-13-sCD146 variant) in patient serum are a product of long-term vascular stress (2).Unlike acute inflammation, in the chronic course of SSc, high concentrations of sCD146 not only indicate vascular loss but may also act as a pre-fibrotic factor, inducing endothelial cells to lose their polarity and transform into fibroblasts capable of secreting collagen (i.e., the EndoMT process) (39). This mechanism closely links early “vascular lesions” with mid-to-late “tissue and skin fibrosis,” explaining why sCD146 levels can predict the severity of skin involvement (40, 69).

#### 3.3.4 Systemic vasculitis

In active small-vessel vasculitis (such as ANCA-associated vasculitis), sCD146 exhibits a unique “non-linear” expression pattern. During highly active pathological phases, due to necrotizing inflammation of the vascular wall, the instantaneous production of sCD146 is extremely high; however, these free proteins rapidly bind to locally activated macrophage receptors or enter the interstitial space during severe exudation. This “local high demand/consumption” can lead to a decrease in free sCD146 detected in peripheral blood. When the disease enters remission and the endothelium begins to repair, this consumption effect disappears, and sCD146 levels return to baseline. This “paradoxical” decrease is an important biochemical clue for clinically determining that vasculitis has entered a high-risk, extremely active phase (70).

#### 3.3.5 Systemic lupus erythematosus

Among systemic autoimmune diseases, sCD146 also demonstrates a sensitive monitoring capability for microvascular endothelial damage. In the pathological progression of systemic lupus erythematosus (SLE), circulating sCD146 levels show a significant positive correlation with the Systemic Lupus Erythematosus Disease Activity Index (SLEDAI) (71).Studies have found that abnormally elevated levels of sCD146 not only indicate the activation and apoptosis of vascular endothelial cells under immune complex deposition but may also participate in mediating leukocyte transendothelial migration, thereby exacerbating local inflammatory damage in organs (such as in lupus nephritis). This makes sCD146 a promising biochemical marker for assessing the activity of SLE vasculitis.

A detailed summary of the clinical translational significance, cohort evidence, and potential targeted interventions for sCD146 in neurological and autoimmune disorders is presented in **Table 2C**.

**Table 2C:** Summary of sCD146 Associations, Clinical Trials, and Interventions in neurology and autoimmunity.

Disease category	Disease name	Core molecular mechanism	Clinical translation significance	Clinical trials (cohort size/strength of evidence)	Potential interventions/target drugs	References
Neurology and Autoimmunity	Multiple sclerosis (MS)	Binds to laminin 411, guiding Th17 cells across the blood-brain barrier (BBB).	Indicates the intensity of neuroinflammation and the extent of central barrier damage.	Large-scale cohort of 823 patients (including 562 cases of neuroinflammation).	Anti-MCAM monoclonal antibody (blocks laminin binding).	(31–33)
	Anti-NMDAR encephalitis	Mediates early changes in BBB permeability, triggering a local inflammatory storm.	Real-time mapping of intracerebral inflammatory activity to aid in treatment stratification.	Prospective observation: 3-month follow-up of 23 patients, correlated with mRS scores.	Enhanced immunosuppressive intervention.	(38)
	Systemic lupus erythematosus (SLE)	Indicates microvascular damage and endothelial cell apoptosis during active disease.	Associated with SLEDAI scores and predictive of organ vascular involvement.	Cross-sectional analysis: Comparison of 51 female SLE patients in the active phase.	Immunosuppression combined with vascular protection.	(71)
	Systemic sclerosis (SSc)	The I5–13 variant induces endothelial-mesenchymal transition (EndoMT) and fibrosis via the Wnt pathway.	Predicts worsening skin sclerosis and fatal pulmonary fibrosis.	Analysis of a cohort of 117 SSc patients and validation in animal models.	Wnt signaling inhibitor.	(39, 40, 69)

### 3.4 Obstetric, reproductive, renal, and other disorders: disruptors of the early life and multiorgan microenvironment

sCD146 plays a critical regulatory role in vascular remodeling at the maternal-fetal interface, embryonic development, and microcirculatory homeostasis of the renal and respiratory systems (34). Upon exposure to hypoxia, metabolic stress, or systemic inflammatory storms, the abnormal release of sCD146 not only serves as a marker of endothelial damage but also directly contributes to tissue destruction and developmental arrest.

#### 3.4.1 Preeclampsia

Normal placental development depends on the successful remodeling of uterine spiral arteries by extra-villus trophoblast (EVT) cells. Studies have confirmed that, at the abnormal maternal-fetal interface in preeclampsia, ischemia-hypoxia leads to the massive release of sCD146, which acts as a “competitive ligand inhibitor. “It binds to galectin-1 (Gal-1) with extremely high affinity in the interstitial space (35), blocking the normal interaction between Gal-1 and receptors on the surface of EVTs, resulting in shallow trophoblast invasion and widespread placental hypoperfusion. Prospective pilot studies have shown that the combined assessment of the PlGF/sCD146 ratio provides a strong predictive tool for screening severe PE in high-risk pregnant women (36).

#### 3.4.2 Recurrent miscarriage

Similar to the pathogenesis of PE, early pregnancy failure is also closely associated with vascular risk factors. Abnormal levels of sCD146 in circulation not only indicate maternal systemic endothelial dysfunction, but its excessive accumulation at the maternal-fetal interface also strongly inhibits trophoblast kinetics and disrupts early embryonic implantation-related vascular remodeling, serving as a crucial biochemical window for identifying potential immune/vascular pathologies underlying unexplained recurrent miscarriage (72).

#### 3.4.3 Bronchopulmonary dysplasia (pediatrics/development)

The pathological role of sCD146 also profoundly influences the developmental trajectory of newborns (73). As a severe challenge facing extremely low birth weight preterm infants, the core pathology of BPD is arrested alveolar development. Under stress conditions involving prolonged exposure to high oxygen levels or hypoxia, the CD146-HIF-1α signaling axis is abnormally activated in the lung tissue of preterm infants. The massive release of sCD146 and the upregulation of its membrane receptors directly impede the migration and repair of alveolar epithelial cells, leading to the arrest of alveolar development. This provides a novel molecular perspective for lung protection and targeted gene intervention in preterm infants.

#### 3.4.4 IVF implantation failure

In the *in vitro* fertilization (IVF) microenvironment, sCD146 has been identified as a core component of the early embryonic secretome (74). However, elevated levels of sCD146 in embryo culture supernatants often reflect a state of “metabolic stress” in the embryo *in vitro*. Excessive secretion of sCD146 disrupts the microenvironmental homeostasis of the endometrial “implantation window.” A large retrospective validation study involving 222 embryos demonstrated that implantation success rates plummeted significantly when the sCD146 concentration in the culture supernatant exceeded 1,164 pg/mL. This makes it an excellent quantitative criterion for the non-invasive selection of high-quality single embryos in assisted reproduction.

#### 3.4.5 Acute and chronic kidney disease

Renal microvessels are extremely sensitive to metabolic toxicity. In diabetic nephropathy (DN) or acute kidney injury (AKI), Wnt5a directly binds to sCD146, strongly activating the downstream JNK/SNAI1 pathway, which drives pathogenic epithelial-mesenchymal transition and inflammatory responses in renal tubules (75, 76). Clinical cohorts demonstrate that as the filtration barrier is compromised, sCD146 exhibits a synchronous abnormal elevation in both blood and urine, and its value in identifying subclinical tubular damage significantly surpasses that of traditional urinary microalbumin testing (77, 78).

#### 3.4.6 Kidney transplant rejection

In allogeneic kidney transplantation, the recipient’s immune system attack on the donor vascular endothelium triggers a “cascade-like” release of sCD146 (79). A validation analysis in transplant patients demonstrated that plasma sCD146 levels surge rapidly during hyperacute and acute rejection (optimal cutoff value: 75.64 ng/ml), with a clinical warning window that significantly precedes the deterioration of serum creatinine levels, making it the “gold standard” for identifying acute immune-mediated damage to the graft endothelium (80).

#### 3.4.7 Others

##### 3.4.7.1 Chronic obstructive pulmonary disease

Harmful exposures such as cigarette smoke extract (CSE) induce the shedding of CD146 from the surface of pulmonary microvascular endothelial cells, leading to barrier disruption and macrophage infiltration. Elevated sCD146 in plasma and bronchoalveolar lavage fluid from COPD patients is an independent risk factor reflecting impaired airway endothelial integrity and acute exacerbations of COPD (AECOPD) (81–83).

##### 3.4.7.2 COVID-19

Severe COVID-19 is essentially a systemic endothelialitis triggered by a cytokine storm. Studies have found that excessive matrix metalloproteinase-1 (MMP-1) in critically ill hospitalized patients excessively hydrolyzes endothelial surface proteins, leading to extreme dysregulation of markers such as sCD146.The explosive release of sCD146 precisely correlates with virus-induced widespread vascular damage and capillary leakage throughout the body, and is closely associated with patient mortality and clinical severity (84).

The clinical associations, diagnostic cohorts, and corresponding therapeutic strategies concerning sCD146 in reproductive, developmental, renal, and other multiorgan microenvironmental disorders are comprehensively summarized in **Table 2D**.

**Table 2D:** Summary of sCD146 associations, clinical trials, and interventions in other disorders.

Disease category	Disease name	Core molecular mechanism	Clinical translation significance	Clinical trials (cohort size/strength of evidence)	Potential interventions/target drugs	References
Other	Preeclampsia (PE)	Inhibiting Gal1 to disrupt the interaction between trophoblast cells and spiral arteries.	Early screening for severe PE and risk of placental hypoperfusion.	Prospective pilot study: Dynamic monitoring of the PlGF/sCD146 ratio in 115 pregnant women.	sCD146 neutralizing antibodies.	(35, 36)
	Recurrent Miscarriage (RM)	Interferes with vascular remodeling at the maternal-fetal interface and inhibits the depth of invasion.	Identifies vascular risk factors for early pregnancy loss.	Retrospective case-control study: Analysis of women with two or more unexplained miscarriages.	Immunomodulation/Low-molecular-weight heparin.	(72)
	BPD (Pediatrics/Development)	Dysregulation of the CD146-HIF-1α axis leads to arrested alveolar development.	Predicting long-term lung development trajectories in very low birth weight preterm infants.	Clinical exploration: Comparison of oxidative stress markers between preterm and term infants.	Precision regulation of the CD146 gene.	(73)
	IVF implantation failure	Disruption of the homeostasis of the endometrial “implantation window.”	Non-invasive evaluation criteria for assisting in the selection of high-quality embryos.	Retrospective validation (n=222 embryos): cutoff value <1164 pg/mL.	Optimization of embryo culture medium formulation.	(74)
	Acute and Chronic Kidney Disease (AKI/DN)	Wnt5a activates JNK signaling to drive renal tubular inflammation.	Identifies subclinical tubular injury better than microalbumin.	Prospective cohort: Follow-up of 159 patients with DN.	Wnt5a antagonist (Box 5).	(75–78)
	Kidney transplant rejection	Indicates that the graft endothelium is undergoing acute immune damage.	Ultra-early warning of rejection preceding changes in serum creatinine.	Retrospective analysis: 56 patients with biopsy-confirmed rejection.	Early intensive anti-rejection intervention.	(79, 80)
	Other (COPD/COVID)	Markers of impaired endothelial integrity and barrier disruption.	Associated with disease severity and vasculitis activity.	Clinical cohorts: Significantly elevated in critically ill hospitalized COVID-19 patients.	Endothelial protection/MMP-1 inhibition.	(81–84

### 3.5 Stem cell biology, tissue engineering, and regenerative medicine

In addition to its roles in pathology, the CD146/sCD146 axis has emerged as a crucial regulatory network in stem cell biology and regenerative medicine.

#### 3.5 .2Stem cell biology and niche regulation

CD146 is established as a defining marker for highly clonogenic multipotent mesenchymal stromal cells (MSCs) and pericytes. Evidence suggests that CD146-positive MSCs exhibit enhanced stemness, secretory profiles, and immunomodulatory properties compared to their negative counterparts (85, 86). Within the bone marrow microenvironment, perivascular CD146+ cells serve as essential components of the hematopoietic stem cell (HSC) niche, critically regulating HSC maintenance and mobilization (87). Furthermore, sCD146 acts as a potent paracrine factor. Preclinical models indicate that it enhances the survival, mobilization, and functional engraftment of endothelial progenitor cells (EPCs) and potentially very small embryonic-like stem cells (VSELs) during post-ischemic tissue rescue (88).

#### 3.5.2 Regenerative medicine and cell-free therapies

In the context of cell-free regenerative strategies, sCD146-enriched extracellular vesicles and exosome biology have garnered significant attention. Preclinical studies suggest that exosome-mediated delivery of CD146 signaling can accelerate tissue repair mechanisms by promoting local angiogenesis, modulating the inflammatory microenvironment, and facilitating the rescue of apoptotic endothelial cells in ischemic tissues (20).

#### 3.5 .3Tissue engineering and vascularized biomaterials

A major hurdle in tissue engineering is establishing functional microvascular networks within engineered constructs. Recent early-phase translational research has actively explored the integration of the sCD146 signaling axis into angiogenic scaffold design and engineered vascular grafts. Preliminary evidence suggests that functionalizing biomaterial surfaces with recombinant sCD146, or utilizing co-culture strategies with CD146-expressing pericytes within 3D matrices, may significantly accelerate *in vivo* endothelialization strategies (89). These approaches hold theoretical promise for improving the long-term patency and structural integration of vascularized biomaterials; however, extensive *in vivo* validation and safety assessments remain to be fully established before advancing to clinical application.

## 4 Clinical translation of sCD146—diagnostic applications and therapeutic potential

Following the identification of sCD146’s molecular role in multisystem diseases, current clinical translation research is focused on developing it as a standard biochemical diagnostic tool and a novel therapeutic target.

### 4.1 Clinical diagnosis and prognosis assessment: application value as a circulating biomarker

The stability of sCD146 in body fluids and its close correlation with disease activity make it a highly promising “liquid biopsy” marker (5, 90–93).

#### 4.1.1 Precision stratification and congestion monitoring in heart failure

In clinical practice, the greatest advantage of sCD146 lies in its high specificity for “tissue congestion” (94). Although constrained by the heterogeneity of detection antibodies, clinical evidence derived from studies utilizing specific standardized ELISA kits demonstrates that the diagnostic cut-off values of sCD146 indicative of congestion in patients with acute and chronic heart failure typically range from 250 to 600 ng/mL. Compared to NT-proBNP, which is susceptible to the effects of age, renal function, and obesity, sCD146 exhibits a higher area under the receiver operating characteristic curve (AUC) in assessing systemic venous congestion (95). Clinicians can use its dynamic changes to assess the therapeutic response to diuretics: a sustained decline in sCD146 levels typically indicates a genuine improvement in hemodynamic status (53, 96). Crucially, compared to myocardial stretch markers (NT-proBNP) or remodeling and fibrosis markers (such as ST2, Galectin-3, and GDF-15), sCD146 provides unique orthogonal information by specifically reflecting microvascular endothelial tension and systemic venous congestion, thereby placing it in a distinct and complementary position within the broader heart failure biomarker landscape.

#### 4.1.2 Monitoring tumor drug resistance and risk prediction

In oncology, the translational value of sCD146 lies in “early efficacy assessment” (97). For example, in glioblastoma patients receiving anti-angiogenic therapy (such as bevacizumab), abnormally elevated serum sCD146 concentrations often precede tumor progression as demonstrated by MRI imaging. Furthermore, by establishing multi-marker combination models (e.g., sCD146 combined with CEA or PSA), the sensitivity of early screening for lung cancer, prostate cancer, and other cancers can be significantly improved, providing a basis for developing personalized treatment plans (25, 48, 98–100).

#### 4.1.3 Monitoring the course of disease and evaluating treatment efficacy in anti-NMDAR encephalitis

Impaired blood-brain barrier (BBB) integrity is a key pathophysiological feature of anti-N-methyl-D-aspartate receptor (NMDAR) encephalitis, directly affecting the penetration of pathogenic antibodies into the central nervous system. Recent clinical data show that sCD146 levels in the cerebrospinal fluid (CSF) of patients with acute anti-NMDAR encephalitis are significantly elevated compared to controls and are upregulated in tandem with the expression levels of pro-inflammatory cytokines (such as TNF-α, IL-6, and IL-10).More importantly, CSF sCD146 concentration showed a significant positive correlation with the Modified Rankin Scale (mRS) score, which reflects the severity of neurological damage. Although sCD146 levels in patients’ CSF decreased significantly during follow-up 3 months after receiving effective treatment, they still differed statistically from healthy baseline levels. This suggests that CSF sCD146 not only accurately reflects early intracerebral inflammatory storms and the extent of BBB damage but also serves as a powerful biomarker for dynamically monitoring disease activity and optimizing early clinical treatment decisions (101).

#### 4.1.4 Early warning of obstetric and renal complications

In the screening for preeclampsia, the ratio of sCD146 to PlGF (placental growth factor) has been explored as a composite indicator for predicting the risk of severe disease (102, 103). In the field of kidney transplantation, a surge in serum sCD146 within 24–48 hours post-transplant is considered an ultra-early biomarker of acute rejection, with a warning window that precedes traditional serum creatinine testing (78, 92, 104, 105).

#### 4.1.4 Sickle cell disease

Sickle cell disease (SCD) is a severe hemolytic disorder characterized by recurrent episodes of painful vascular occlusion, leading to ischemia-reperfusion pathophysiology and subsequent tissue damage. Oxidative stress, endothelial cell activation, inflammation, and vascular dysfunction are central to this process. Data suggest that endothelial activation markers such as sCD146 may aid in evaluating curative and non-curative therapies for SCD patients (106).

#### 4.1.5 Inflammatory myopathies

Clinical studies have confirmed that in the peripheral circulation of patients with polymyositis (PM), levels of soluble adhesion molecules (with sCD146 as the primary representative) that reflect endothelial cell junctional integrity exhibit a specific, abnormal surge. More critically, sCD146 displays distinctly different circulatory expression profiles between PM and dermatomyositis (DM).This highly heterogeneous “molecular fingerprint” not only profoundly reflects the fundamental differences in the underlying pathophysiological mechanisms (particularly microvascular stress and endothelial injury pathways) between the two myopathy subtypes, but also suggests that sCD146 holds promise as a highly sensitive discriminatory biomarker to assist clinicians in precisely distinguishing the microvascular involvement characteristics of PM and DM (107).

#### 4.1.6 Communication disruption in the “placenta-brain” axis under physicochemical stress and developmental warning signs

sCD146 also plays an irreplaceable role in inter-organ communication during early embryonic development. Recent preclinical investigations suggest that sCD146 may act as a key endocrine messenger that maintains the “placenta-brain” pro-angiogenic axis. Under normal physiological conditions, CD146, highly expressed in placental endothelial cells, is shed and enters the fetal circulation in large quantities in a soluble form, remotely coordinating local angiogenesis in the fetal brain. However, when subjected to intense physicochemical stressors such as prenatal alcohol exposure (PAE), this inter-organ communication undergoes a “profound signaling disruption. “PAE directly leads to the depletion of sCD146 release from the placenta, severing the distal angiogenic signals; this signaling deprivation not only causes a decrease in cortical vascular density and spatial disorganization in the fetal brain but also secondarily disrupts the perivascular microenvironment, resulting in impaired oligodendrocyte development. At the clinical translation level, the PAE-induced abnormal sCD146 expression gradient—characterized by “low peripheral and high central” levels—holds significant diagnostic value. The significant decrease in peripheral blood sCD146 concentration holds promise as a sensitive biological indicator for monitoring occult neonatal neurovascular defects (such as fetal alcohol spectrum disorders), thereby securing a valuable time window for early neuroprotective interventions before and after birth (38).

### 4.2 Therapeutic potential and translational medicine: multidimensional targeted intervention strategies for the sCD146 signaling axis

Given the central role of sCD146 in angiogenesis (108), tumor metastasis (109), inflammatory infiltration, and tissue fibrosis (110), the development of therapeutic strategies targeting this signaling axis has become a hot topic in translational medical research (111).Compared to traditional single-target interventions, current therapeutic strategies are evolving toward a three-dimensional model that blocks the entire signaling pathway at the “upstream, midstream, and downstream” levels (112, 113).

#### 4.2.1 Upstream intervention: “cutting off the supply at the source” by inhibiting protein hydrolysis and detachment

Blocking the conversion of membrane-bound CD146 into its soluble form is a fundamental strategy for reducing the sources of pathological sCD146. This intervention focuses on inhibiting the key metalloproteinases ADAM10 and TACE (ADAM17), which mediate endothelial cell detachment. Studies have shown that the use of highly selective ADAM10 inhibitors (such as GI254023X) and broad-spectrum metalloproteinase inhibitors (such as Marimastat) can effectively reduce the loss of cell surface CD146. This not only lowers the baseline levels of sCD146 in the microenvironment and circulatory system at the source but also alleviates the early endothelial stress mediated by sCD146.

However, evaluated from the rigorous perspective of translational medicine, the “upstream strategy” of inhibiting enzymatic shedding currently faces formidable barriers regarding clinical toxicity. Broad-spectrum matrix metalloproteinase inhibitors (MMPIs), represented by Marimastat, although demonstrating impeccable logic in theory and preclinical models, suffered widespread attrition in early Phase III clinical trials for solid tumors. The fundamental cause of this failure stems from the exceptionally high structural homology within the catalytic active centers (zinc-binding domains) between matrix metalloproteinases and the ADAM family, precipitating severe non-specific “off-target effects.” Clinically, this directly induced dose-limiting musculoskeletal syndrome (MSS), characterized by debilitating joint stiffness, myalgia, and inflammation. Moreover, ADAM10 and TACE (ADAM17) execute indispensable physiological “housekeeping” functions across multiple systemic networks (e.g., mediating Notch signaling and physiological TNF-α shedding). Consequently, systemic pharmacological blockade of these enzymes severely compromises normal physiological homeostasis (114, 115).

#### 4.2.2 Midstream interventions: ligand neutralization and physical interception

Directly “capturing” free sCD146 in the extracellular space to prevent it from binding to target receptors is currently the approach with the fastest clinical translation and highest specificity.

##### 4.2.2.1 Specific neutralizing antibodies

Several monoclonal antibodies targeting sCD146 functional epitopes have been developed (e.g., AA98, M2J-1, and the humanized monoclonal antibody Mucizumab).Among these, M2J-1 can precisely recognize and envelop the active site of sCD146, blocking its physical interaction with AMOT or VEGFR2, thereby significantly inhibiting angiogenesis and metastasis in solid tumors such as melanoma in preclinical models (41).

##### 4.2.2.2 Decoy receptors

The development of “decoy” molecules (such as free fragments mimicking the extracellular domain of the target receptor) using recombinant protein technology to preferentially bind sCD146 in the blood is another highly promising competitive interception strategy.

However, considering the pivotal physiological role of sCD146 in vascular repair under peripheral ischemic conditions (e.g., PAD), the future clinical application of sCD146 neutralizing antibodies in anti-tumor regimens mandates rigorous surveillance for potential off-target cardiovascular risks, such as impaired wound healing or the exacerbation of occult ischemia.

#### 4.2.3 Downstream interventions: receptor antagonism and intracellular signal blockade

To address the abnormal signaling cascades triggered by sCD146 binding to its receptor, multi-tiered precise prevention and control can be achieved by blocking core intracellular signaling pathways in target cells:

PI3K/AKT signaling pathway inhibitors: Studies have confirmed that sCD146 relies on the activation of the downstream PI3K/AKT signaling cascade to maintain abnormal endothelial cell survival and compensatory angiogenesis. Therefore, targeted inhibition of this core pathway can effectively disrupt sCD146-mediated pro-proliferative signals. This intervention strategy is particularly suitable for combating early bypass resistance to VEGF therapy in solid tumor models (15, 49).

MAPK/ERK inhibitors (e.g., Trametinib): By curbing the “synthetic” transformation of smooth muscle cells driven by sCD146-mediated intercellular crosstalk, these agents hold promise as new intervention targets for pulmonary hypertension-associated vascular remodeling (116).

Wnt inhibitors (e.g., WNT-974): By blocking the Wnt/β-catenin signaling axis, these agents fundamentally curb endothelial-mesenchymal transition (EndoMT) in the fibrosis process driven by a specific variant (I5-13-sCD146) (117).

**Figure 3:**
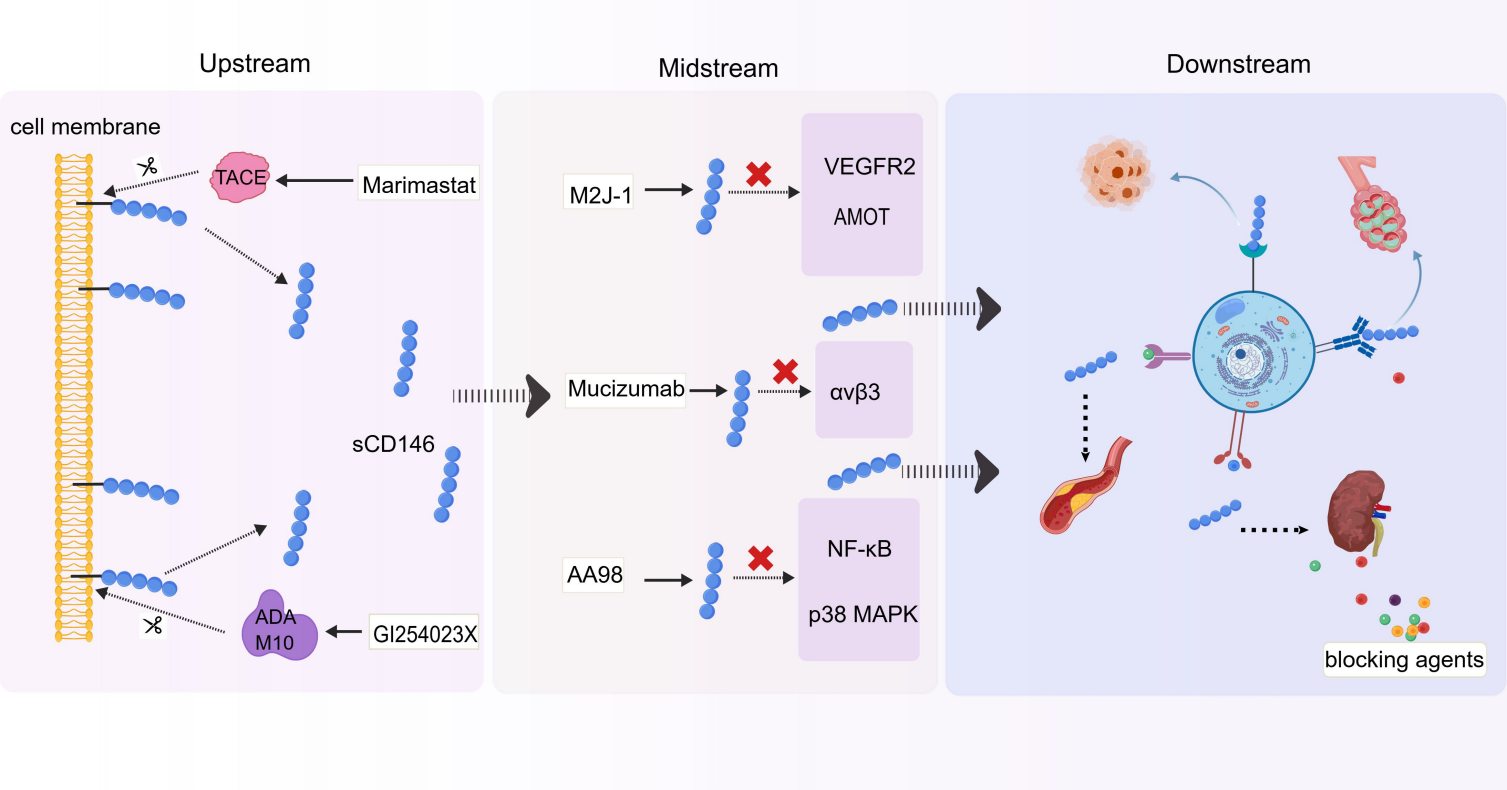
Comprehensive multidimensional targeted intervention and therapeutic strategies against the sCD146 pathogenic signaling axis. Based on the sCD146-mediated cascade, current translational interventions can be categorized into a three-dimensional framework: **(A)** Upstream inhibition: Utilizing ADAM10/TACE inhibitors (e.g., GI254023X) to prevent the pathological enzymatic shedding of sCD146 at the source; **(B)** Midstream physical interception: Deploying specific monoclonal antibodies (e.g., M2J-1, Mucizumab) or decoy receptors to precisely neutralize free sCD146 in the extracellular space, thereby severing abnormal intercellular crosstalk; **(C)** Downstream pathway blockade: Employing small-molecule kinase inhibitors to target core intracellular pathogenic networks overactivated by sCD146 (e.g., PI3K/AKT, MAPK/ERK, or Wnt pathways). Furthermore, the local application of recombinant sCD146 protein (bottom right) demonstrates endogenous regenerative potential in promoting post-ischemic repair in peripheral artery disease (PAD).

To provide a more intuitive representation of the logical framework of this full-pathway blockade, **Figure 3** systematically illustrates a “three-dimensional” overview of intervention strategies targeting the sCD146 pathogenic axis.

It is worth noting that sCD146 possesses bidirectional biological properties. In contrast to the aforementioned blockade strategies, in peripheral artery disease (PAD) or acute myocardial infarction, sCD146 is regarded as an “endogenous repair protein. “Local injection of recombinant human sCD146 protein, or local intervention using endothelial progenitor cells that highly express sCD146, can significantly induce the formation of new collateral circulation in damaged areas, providing an endogenous repair pathway for patients with severe ischemia.

### 4.3 Overcoming clinical translation bottlenecks: breakthrough strategies toward precision medicine

A critical appraisal of the current literature reveals that while preclinical models have elucidated the mechanistic causation of sCD146 in disease progression, a significant portion of the clinical utility discussed remains largely associative. One of the major barriers to clinical adoption is the lack of large-scale, independent validation cohorts and standardized Receiver Operating Characteristic (ROC) analyses to firmly establish sensitivity and specificity across diverse populations. Furthermore, dedicated interventional clinical trials specifically targeting the sCD146 axis remain exceedingly rare.

To bridge the “Valley of Death” between basic laboratory discoveries of sCD146 and real-world clinical applications, future translational medical research must urgently focus on the following three core pathways:

Structure-driven antibody engineering: Dedicated to developing novel monoclonal antibodies that specifically recognize “cryptic epitopes” exposed only after the enzymatic cleavage of free sCD146.This approach aims to precisely neutralize pathogenic sCD146 in circulation while maximizing the preservation of the physiological function of membrane-bound CD146 (mCD146) in maintaining the integrity of the vascular endothelial barrier, thereby effectively avoiding the systemic endothelial toxicity that targeted therapies may cause (6).

Global standardization of detection assays: There is an urgent need to establish a unified system of sCD146 standard reference materials and quality control protocols in collaboration with authoritative international organizations. By eliminating systematic errors between different detection platforms and antibody clones, and establishing a universally accepted clinical diagnostic cutoff in multicenter, large-sample real-world cohorts, this serves as a prerequisite for developing sCD146 into a standardized companion diagnostic (CDx) tool.

Dynamic “spatiotemporal-gated” intervention: Given that sCD146 possesses dual biological properties as both a “driver of tissue damage” and an “endogenous repairer of ischemia,” future clinical decision-making must incorporate considerations of spatiotemporal heterogeneity. Clinically, it is necessary to dynamically track its circulating levels via liquid biopsy to precisely define the “time window” for intervention—for example, decisively blocking its action during the unchecked progression of malignant tumors, while exploring local supplementation strategies during the long-term recovery phase of acute ischemic stroke or myocardial infarction, with the aim of achieving truly personalized precision diagnosis and treatment (118).

### 4.4 Limitations of current clinical evidence

Despite the promising translational potential of the sCD146 signaling axis, several critical limitations must be explicitly acknowledged to provide a balanced perspective. First, the majority of current clinical data are derived from observational and retrospective cohorts, making it challenging to definitively establish causal relationships rather than mere associations. Second, limited interventional validation exists, as dedicated interventional clinical trials specifically targeting the sCD146 axis remain exceedingly rare. Third, significant assay heterogeneity, varying antibody clone specificities, and a lack of global standardization across detection platforms prevent the establishment of universal clinical diagnostic cut-offs. Consequently, it must be explicitly stated that, at present, no FDA- or EMA-approved diagnostic assay or therapeutic intervention specifically targeting sCD146 currently exists. Addressing these barriers through large-scale, prospective, and independent multicenter validation cohorts is essential before sCD146 can be routinely integrated into clinical decision-making.

## 4 Conclusion

In summary, sCD146 has transcended the traditional understanding of it as a metabolic byproduct of endothelial cells and has been established as a key factor mediating angiogenesis, immune infiltration, and the remodeling of the tissue microenvironment. From a “catalyst” of tumor resistance to a “projector” of congestive heart failure, and further to an “interferer” in maternal-fetal developmental abnormalities such as preeclampsia and BPD, precision inhibition of sCD146 demonstrates promising translational prospects across multiple disciplines.

However, it cannot be overlooked that its clinical translation remains in the challenging “deep waters.” The risk of endothelial toxicity associated with systemic blockade, technical barriers in distinguishing between membrane-bound and soluble forms, and the lack of a standardized companion diagnostic (CDx) system all represent gaps that urgently need to be bridged. Furthermore, given that sCD146 possesses the “double-edged sword” properties of both tissue damage and ischemic repair, future clinical intervention strategies must account for the “spatiotemporal heterogeneity” of the disease.

Looking ahead, the key to overcoming these bottlenecks lies in: (1) accelerating the establishment of cross-center consensus on sCD146 clinical quality control and cutoff values; (2) developing highly specific antagonists targeting only specific pathological variants or binding sites; and (3) exploring combination regimens of sCD146 blockers with immunotherapies or conventional anti-angiogenic drugs. It is foreseeable that, with the continued unraveling of its multidimensional molecular network, sCD146 will inevitably evolve from a laboratory biochemical marker into a powerful clinical tool for the precision diagnosis and treatment of complex diseases in the near future.

## Supplementary material

Supplementary Figure 1 Proteolytic shedding mechanism of sCD146. Triggered by signals such as calcium (Ca2+) influx, membrane-bound CD146 is recognized and cleaved by matrix metalloproteinases (predominantly ADAM-10 and TACE). Its extracellular domain is shed, generating free sCD146 that is released into the extracellular microenvironment.
