# Supplementary figures and images for: Biological properties of soluble CD146 and its role and mechanisms in disease progression
Source: Front Immunol. 2026 Jul 20;17:1892968. doi: 10.3389/fimmu.2026.1892968 (PMC13429764; doi:10.3389/fimmu.2026.1892968)

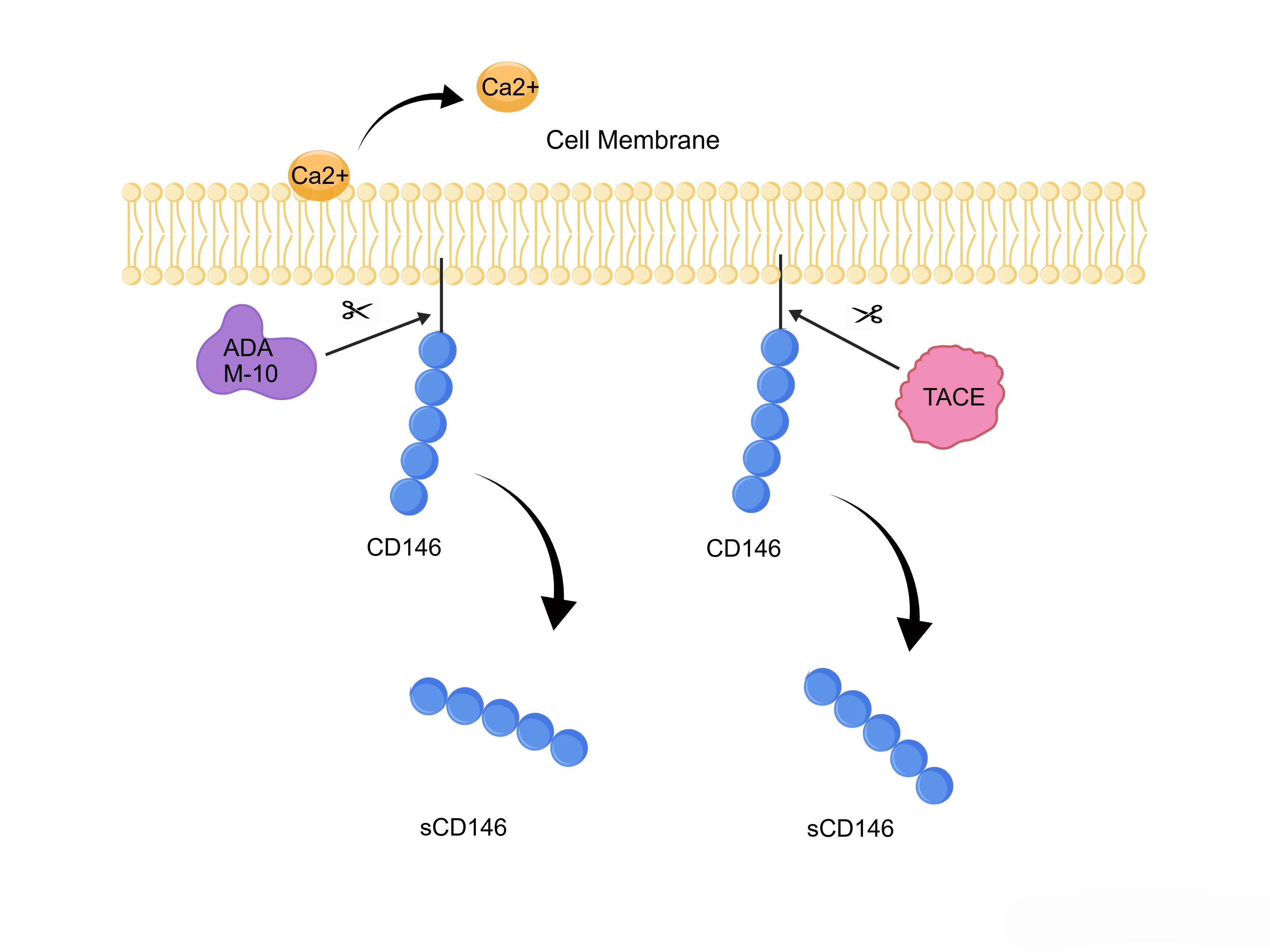

Supplement: Supplementary Figure 1 — Proteolytic shedding mechanism of sCD146. Triggered by signals such as calcium (Ca2+) influx, membrane-bound CD146 is recognized and cleaved by matrix metalloproteinases (predominantly ADAM-10 and TACE). Its extracellular domain is shed, generating free sCD146 that is released into the extracellular microenvironment. [file Image1.jpeg]
